# Impact of the COVID-19 pandemic on student experiences during rural placements in Australia: findings from a national multi-centre survey

**DOI:** 10.1186/s12909-022-03927-1

**Published:** 2022-12-09

**Authors:** Priya Martin, Matthew McGrail, Jordan Fox, Remo Ostini, Zelda Doyle, Denese Playford, Jessica Beattie, Vivian Isaac, Lara Fuller, Penny Allen, Srinivas Kondalsamy-Chennakesavan

**Affiliations:** 1 Rural Clinical School, Faculty of Medicine, The University of Queensland, Locked Bag 9009, Toowoomba, QLD 4350 Australia; 2 Rural Clinical School, School of Medicine, University of Notre Dame, Sydney, Australia; 3 Rural Clinical School of Western Australia, Medical Schools of UWA and Notre Dame, The University of Western Australia, Perth, Australia; 4 Rural Community Clinical School, School of Medicine, Deakin University, Geelong, Australia; 5 College of Medicine and Public Health, Rural and Remote Health SA, Flinders University, Adelaide, Australia; 6 Rural Clinical School, College of Health and Medicine, The University of Tasmania, Hobart, Australia

**Keywords:** Rural placements, Clinical placements, COVID-19 pandemic, Medical education

## Abstract

**Background:**

The aim of this national study was to explore the learning experiences of Australia’s medical students who trained rurally during the COVID-19 pandemic in 2020.

**Methods:**

A cross-sectional, national multi-centre survey was conducted in 2020, through the Federation of Rural Australian Medical Educators (FRAME). Participants were medical students who had completed an extended Rural Clinical School (RCS) training placement (≥ 12 months). A bespoke set of COVID-19 impact questions were incorporated into the annual FRAME survey, to capture COVID-19-related student experiences in 2020. Pre-pandemic (2019 FRAME survey data) comparisons were also explored.

**Results:**

FRAME survey data were obtained from 464 students in 2020 (51.7% response rate), compared with available data from 668 students in 2019 (75.6% response rate). Most students expressed concern regarding the pandemic’s impact on the quality of their learning (80%) or missed clinical learning (58%); however, students reported being well-supported by the various learning and support strategies implemented by the RCSs across Australia. Notably, comparisons to pre-pandemic (2019) participants of the general RCS experience found higher levels of student support (strongly agree 58.9% vs 42.4%, *p* < 0.001) and wellbeing (strongly agree 49.6% vs 42.4%, *p* = 0.008) amongst the 2020 participants. Students with more than one year of RCS experience compared to one RCS year felt better supported with clinical skills learning opportunities (*p* = 0.015) and less affected by COVID-19 in their exam performance (*p* = 0.009).

**Conclusions:**

This study has provided evidence of both the level of concern relating to learning quality as well as the positive impact of the various learning and support strategies implemented by the RCSs during the pandemic in 2020. RCSs should further evaluate the strategies implemented to identify those that are worth sustaining into the post-pandemic period.

## Introduction

Medical programs in universities across the world have had to respond to the COVID-19 pandemic by enacting changes to curricular delivery, clinical placements, and examinations [1–3]. It is envisaged that some of the learnings and program improvements induced by forced changes will be sustained into the post-pandemic period, as part of curricular reforms [3].

Internationally, disruptions to clinical training for medical students have stemmed from a climate of uncertainty around the risks of the COVID-19 pandemic leading to discontinuation of clinical placements either by the placement site or university [4, 5]. This discontinuation of placements meant that students were unable to practice applied skills or gain experience interacting with patients [5, 6]. Due to the stress of the pandemic coupled with significant restrictions to clinical placements, medical students world-wide felt the effects of the pandemic, which manifested as mental health and wellbeing challenges, significant disruptions to their learning and concerns about timely graduation and career progression [1, 7]. Information on challenges experienced by medical and nursing students undertaking rural placements in Canada [8] and nursing and allied health students undertaking clinical placements across rural Australia [9] has emerged. In contrast, limited information is currently available about the experience of medical students completing clinical placements in rural Australia.

**Fig. 1:**
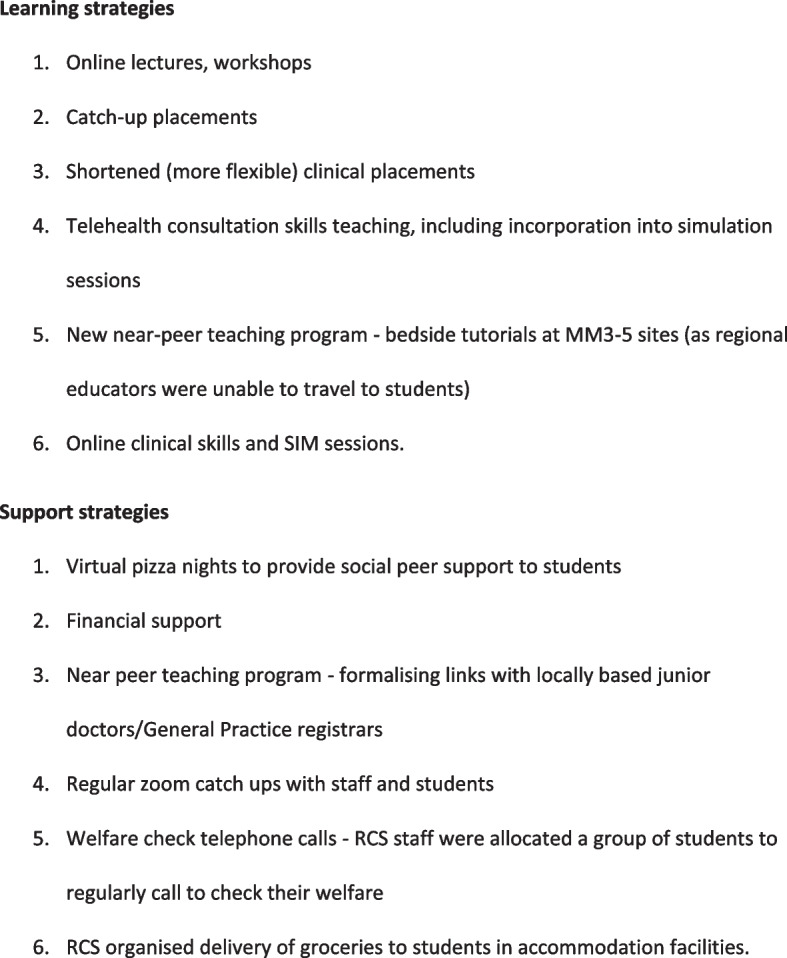
Additional support and learning strategies implemented by RCSs during the pandemic

Evidence of the value of rural medical education is growing, particularly to supplying the future rural medical workforce [10–12]. The continuation of rural clinical placements for medical students was not immune to the challenges induced by the pandemic. The Federation of Rural Australian Medical Educators (FRAME) [13] a peak body representing 19 universities with Rural Clinical Schools (RCS) funded by the Australian Government Rural Health Multidisciplinary Training (RHMT) Program [14], provided a platform for Australian rural medical educators to jointly problem-solve and work towards mitigating the adverse effects of the pandemic. Rural programs remained agile and creative, designing, and implementing a range of targeted initiatives to sustain the rural clinical placements. Adaptations implemented included online delivery of lectures using problem-based learning approaches, virtual student support groups, dedicated financial and administrative support from universities, shortened clinical placements, catch-up placements, student exposure to telehealth, and delaying and providing concessions to assessments (see examples in Fig. 1). Student experiences and the impact of these adaptations on student learning need to be investigated to inform future learning and support strategies [1].

Several concerns have been raised about the consequences of missed learning opportunities during clinical placements undertaken throughout the pandemic, particularly once students transition to become junior doctors [15]. It has been widely acknowledged that lack of adequate in-person experiences can negatively impact professional skills such as teamwork, communication, building rapport, empathy, and professionalism, in addition to clinical skills and knowledge [8, 16, 17]. These adverse impacts could be worse for medical students based in rural areas because of professional and social isolation and poor technical infrastructure as highlighted by recent studies from countries including Russia [18], India [19], and Canada [8], compounding the challenges already faced in rural areas.

To date, there is a distinct lack of evidence on the extent and nature of disruptions caused by the pandemic on rural student’s learning, and their perceptions of the learning and support strategies implemented. This information is essential to not only guide the development of targeted strategies that can be implemented to support the emerging medical workforce, but also to guide rural medical training into the post-pandemic period, in relation to curricular and learning adaptations and approaches. With this background in mind, this national multi-centre study aimed to explore the learning experiences of Australia’s medical students who trained rurally during the COVID-19 pandemic. Specifically, we sought to investigate the impact of the COVID-19 pandemic on the training quality of medical students and evaluate how effective the various supports and adjusted learning measures were in supporting students during this challenging time.

## Methods

A cross-sectional quantitative survey, including newly developed questions to assess the impact of the COVID-19 pandemic was administered between June and December 2020 with students from 17 Australian universities (464 respondents). The survey was distributed through FRAME’s annual rural placement evaluation, with invitations distributed to all relevant students on exit from RCS at the end of the placement and in their year of graduation. Each university was responsible for local distribution of invitations and noting the number of eligible participants. Overall response rates were calculated assuming that all eligible participants in each university received an invite. Most students completed the survey towards the end of 2020, although students from two participating RCSs completed the survey in mid-2020 in line with their placements concluding. Participants were medical students who had completed an extended clinical training placement in a rural location of at least one academic year (≥ 12 months), with some students having completed two consecutive years at an RCS.

In addition to FRAME’s standard questions, a bespoke set of COVID-19 impact questions was designed and integrated to capture student experiences in 2020 in relation to the pandemic. These new questions related to their views on learning adaptations and concessions made to assessments as a result of the pandemic, and other learning impacts resulting from the pandemic including on their work-readiness. FRAME’s standard questionnaire includes information relating to the student’s background and demographics, their plans for future medical practice, experience at the RCS and clinical placement environments, and student support and wellbeing. For this study, 2019 FRAME data were additionally used (668 respondents) to provide a pre-pandemic comparison point of student support and wellbeing. A complete copy of the 2019 and 2020 survey instruments can be found on the FRAME website (https://ausframe.org/publications-and-resources/). Ethics approval for this study was obtained through University of Notre Dame Human Research Ethics Committee (ref: 2020-196S).

### Statistical analyses

Descriptive statistics (frequencies and proportions) were calculated, and Chi-Square (Yes/No responses) and Mann–Whitney tests (Likert scale responses) were used for comparisons between groups. Demographic data and questions relating to student support and wellbeing were compared between the 2020 participants and 2019 (pre-pandemic) participants. Likert scales were not collapsed for data analysis but are presented in results tables as positive, negative, and neutral categories (Tables 2 and 3) or as the proportion who answered yes/agree, reported a negative impact, or felt well-supported (Table 4) for ease of interpretation. Comparisons between 2019 and 2020 are reported as strongly agree/strongly disagree, agree/disagree, and all other responses (Table 3) to demonstrate changes in the number of responses at the positive end of the scale. Comparisons were also made between data collected from students having completed their first RCS year and those who have completed more than one RCS year, as well as between participants completing the survey mid-year compared to the end of year. All data were cleaned, coded, and analysed in SPSS version 27.

## Results

FRAME survey data were obtained from 464 students in 2020 (51.7% response rate), compared with available data from 668 students in 2019 (75.6% response rate), however some data are missing due to students not completing all the questions on the survey. Almost 60% (*n* = 266) of the respondents were female and 54% (*n* = 252) had a rural background. For 65% (*n* = 294) of the students, 2020 was their only RCS year. Further descriptive statistics for the 2019 and 2020 cohorts are presented in Table 1.

**Table 1:** Participant demographics

Variable	Response	2019 (N [%])	2020 (N [%])
Gender	Male	278 (43.0)	182 (40.6)
	Female	366 (56.7)	266 (59.4)
	Other	2 (0.3)	0 (0.0)
Age	20–24	362 (57.0)	221 (50.3)
	25–29	198 (31.2)	170 (38.7)
	30 +	75 (11.8)	48 (10.9)
Rural background	No	338 (51.1)	211 (45.6)
	Yes	323 (48.9)	252 (54.4)
Aboriginal/Torres Strait Islander	No	646 (97.9)	451 (97.4)
	Yes	14 (2.1)	12 (2.6)
Already have a health professional qualification	No	595 (90.2)	391 (84.4)
	Yes	65 (9.8)	72 (15.6)
First in family to attend university	No	553 (84.0)	393 (84.9)
	Yes	105 (16.0)	70 (15.1)
Speak a language other than English at home	No	537 (81.5)	378 (81.6)
	Yes	122 (18.5)	85 (18.4)
You and your parents immigrated to Australia	No	415 (62.7)	291 (63.1)
	Yes	247 (37.3)	170 (36.9)
Time of survey completion	Mid-year	Not analysed	64 (14.2)
	End of year	Not analysed	388 (85.8)
RCS year completed	First year at an RCS	Not analysed	294 (65.0)
	Second consecutive year at an RCS year	Not analysed	158 (35.0)

During the pandemic, 88% of respondents always felt safe in clinical training. Of the 33% of students who actively participated in clinical or administrative duties including screening, testing, or treating patients with COVID-19, 78% (*n* = 111) reported always feeling safe. Asked to compare their experience to the beginning of 2020, the majority of respondents (80%) reported a negative impact of COVID-19 on the quality of their learning (specifically on missed learning opportunities, reduced community placements, reduced breadth of cases, and being negatively impacted by travel restrictions). Although more than half (58%) the students were concerned about missed clinical learning, overall students were generally satisfied with adjusted learning methods and changes to assessments (Table 2). No significant changes were noted between the cohorts in relation to their intentions to practice rurally following graduation.

**Table 2:** Satisfaction with adjusted learning and support provided during the pandemic (2020)

Question	No (N [%])	Yes (N [%])	
Participation with COVID-19 patients	292 (67.1)	Clinical: 114 (26.2) Administrative: 29 (6.7)	
Felt safe in clinical training during the COVID-19 pandemic	51 (11.8)	382 (88.2)	
	**Negative**	**Neutral**	**Positive**
COVID-19 impact on quality of learning in clinical placement	347 (80)	53 (12.2)	34 (7.8)
	**Disagree**	**Neutral/Don’t know**	**Agree**
I am concerned about having missed specific clinical learning	125 (28.8)	58 (13.4)	251 (57.8)
I had exposure to an increased breadth of cases	299 (68.9)	78 (18.0)	57 (13.1)
I had more exposure to new models of care	67 (15.5)	55 (12.7)	311 (71.8)
I am worried about my progression into the next year of study	273 (63.0)	65 (15.0)	95 (22.0)
I had more exposure to community placements	274 (63.3)	78 (18.0)	81 (18.7)
Placements/learning opportunities affected by travel restrictions	186 (42.9)	42 (9.7)	205 (47.3)
COVID-19 adversely affected exam performance	107 (24.8)	150 (34.7)	175 (40.5)
	**Dissatisfied**	**Neutral**	**Satisfied**
Satisfaction with teleconferencing of tutoring/learning	99 (22.8)	88 (20.3)	247 (56.9)
Satisfaction with online learning platforms	88 (20.3)	111 (25.6)	235 (54.1)
Satisfaction with self-directed learning	95 (21.9)	134 (30.9)	205 (47.2)
Satisfaction with collaborative learning	86 (19.9)	161 (37.3)	185 (42.8)
Satisfaction with lecture sharing with other universities	104 (24.0)	220 (50.7)	210 (25.3)
Satisfaction with video tutorials	89 (20.6)	136 (31.4)	208 (48.0)
Satisfaction with changes to written assessments	128 (29.7)	NA	303 (70.3)
Satisfaction with changes to case-based learning requirements	126 (29.2)	NA	306 (70.1)
Satisfaction with changes to clinical assessments	141 (32.6)	NA	291 (67.4)
Satisfaction with changes to competency assessments	118 (27.4)	NA	313 (72.6)
**How well do you feel you were supported in the following areas:**	**Poorly supported**	**Neutral/NA**	**Well supported**
Regular communication	52 (12.1)	43 (10.0)	335 (77.9)
Alternatives to clinical work	88 (18.1)	128 (29.8)	224 (52.1)
Q & A opportunities with RCS staff and faculty	55 (12.8)	60 (14.0)	314 (73.2)
Online learning/teaching	45 (10.5)	64 (14.8)	321 (74.7)
Simulation/extra clinical skills learning opportunities	106 (24.6)	75 (17.5)	249 (57.9)
Financial support	78 (18.2)	174 (40.8)	175 (41.0)

Comparisons for student support and wellbeing between cohorts attending RCS training prior to or during the pandemic are provided in Table 3. Students felt better supported academically, financially, and overall, during 2020 compared to 2019 (*P* < 0.001) as well as being more likely to agree that their RCS placement positively impacted their wellbeing (*P* = 0.008). In 2020, students were also less likely to report feeling academically isolated compared to 2019 (*P* = 0.023). In fact, more of the 2020 cohort compared with the 2019 cohort agreed (in the positive direction) to all aspects of support and wellbeing.

**Table 3:** Support and wellbeing comparisons between pre-pandemic (2019) and mid-pandemic (2020) participants

Support and wellbeing comparisons							
**Question**	**2019 (N [%])**			**2020 (N [%])**			**Statistical significance**
	**Strongly agree**	**Agree**	**All other responses**	**Strongly agree**	**Agree**	**All other responses**	
I felt well supported academically by my RCS	298 (47.5)	212 (33.8)	117 (18.7)	271 (60.8)	111 (24.9)	64 (14.3)	*P* < 0.001
I felt well supported financially by my RCS	218 (34.7)	161 (25.6)	250 (39.7)	215 (48.0)	93 (20.8)	140 (31.3)	*P* < 0.001
My RCS informed me of health and counselling services	169 (27.0)	258 (41.1)	200 (31.9)	147 (32.9)	160 (35.8)	140 (31.3)	*P* = 0.132
Overall, I felt well supported by my RCS	266 (42.4)	248 (39.6)	113 (18.0)	264 (58.9)	120 (26.8)	64 (14.3)	*P* < 0.001
My RCS placement impacted positively on my wellbeing	264 (42.4)	203 (32.6)	156 (25.0)	222 (49.6)	139 (31.0)	87 (19.4)	*P* = 0.008
I have a rural-based clinician as a mentor	177 (28.3)	176 (28.1)	273 (43.6)	173 (38.6)	116 (25.9)	159 (35.5)	*P* = 0.003
	**Strongly disagree**	**Disagree**	**All other responses**	**Strongly disagree**	**Disagree**	**All other responses**	
I felt academically isolated during my rural placement	112 (17.9)	186 (29.7)	329 (52.5)	103 (23.0)	137 (30.6)	207 (46.3)	*P* = 0.023
I felt socially isolated during my RCS placement	140 (22.4)	162 (25.9)	324 (51.8)	116 (26.0)	113 (25.3)	218 (48.8)	*P* = 0.312

Students who had completed one year at an RCS were more concerned about progressing to the next year of study (*P* = 0.020) and were more likely to agree that COVID-19 adversely affected their exam performance (*P* = 0.009) than students who had completed more than one year at an RCS (Table 4). Students who had completed just one year at an RCS reported more exposure to community placements (*P* = 0.002) and felt they were less supported in extra clinical skills learning opportunities (*P* = 0.015).

**Table 4:** Support and wellbeing comparisons by timing of survey completion and length of RCS placement

Question	Timing of survey completion comparisons			RCS year comparisons		
	**Yes: Mid-year (N [%])**	**Yes: End of year (N [%])**	**Statistical significance**	**Yes: 1 year (N [%])**	**Yes: > 1 year (N [%])**	**Statistical significance**
Participation with COVID-19 patients	39 (61.9)	104 (28.0)	*P* < 0.001	97 (33.8)	46 (31.0)	*P* = 0.649
Felt safe in clinical training during the pandemic	44 (69.8)	338 (91.4)	*P* < 0.001	97 (33.8)	46 (31.1)	*P* = 0.323
	**Negative impact: Mid-year**	**Negative impact: End of year**		**Negative impact: 1 year**	**Negative impact: > 1 year**	
COVID-19 impact on quality of learning during placement	50 (79.3)	297 (80.1)	*P* = 0.231	232 (80.8)	115 (78.2)	*P* = 0.357
	**Agree: Mid-year**	**Agree: End of year**		**Agree: 1 year**	**Agree: > 1 year**	
I am concerned about having missed specific clinical learning	33 (52.4)	218 (58.7)	*P* = 0.419	176 (61.4)	75 (51.0)	*P* = 0.079
I had exposure to an increased breadth of cases	5 (7.9)	52 (14.0)	*P* = 0.034	47 (16.4)	10 (6.8)	*P* = 0.317
I had more exposure to new models of care	46 (73.0)	265 (71.70)	*P* = 0.140	208 (72.7)	103 (70.1)	*P* = 0.361
I am worried about my progression into the next year of study	6 (9.7)	89 (24.0)	*P* = 0.029	68 (23.8)	27 (18.4)	*P* = 0.020
I had more exposure to community placements	11 (17.7)	70 (18.9)	*P* = 0.806	67 (23.4)	13 (19.6)	*P* = 0.002
Placements/learning opportunities affected by travel restrictions	38 (60.3)	167 (45.1)	*P* = 0.004	137 (47.7)	68 (46.6)	*P* = 0.552
COVID-19 adversely affected exam performance	31 (49.2)	144 (39.0)	*P* = 0.033	127 (44.4)	48 (32.9)	*P* = 0.009
**Level of support:**	**Well supported: Mid-year**	**Well supported: End of year**		**Well supported: 1 year**	**Well supported: > 1 year**	
Regular communication	33 (52.4)	302 (82.3)	*P* < 0.001	221 (78.1)	114 (77.6)	*P* = 0.620
Alternatives to clinical work	26 (41.3)	198 (54.0)	*P* = 0.032	148 (52.3)	76 (51.8)	*P* = 0.162
Q & A opportunities with RCS staff and faculty	35 (55.6)	279 (76.2)	*P* = 0.005	204 (72.4)	110 (74.8)	*P* = 0.161
Online learning/teaching	35 (55.6)	286 (78.0)	*P* < 0.001	209 (73.9)	112 (76.2)	*P* = 0.256
Simulation/extra clinical skills learning opportunities	28 (44.4)	221 (60.2)	*P* = 0.006	154 (54.4)	95 (64.7)	*P* = 0.015
Financial support	14 (22.5)	143 (39.2)	*P* < 0.001	116 (41.5)	59 (40.2)	*P* = 0.598

Students who completed the survey at the end of 2020 reported less participation with COVID-19 patients, and feeling safer in clinical training during the pandemic than those who completed the survey mid-year (*P* < 0.001) (Table 4). Additionally, they reported exposure to an increased breadth of medical presentations overall (*P* = 0.034), though the proportions were low in both groups (14% versus 8% respectively). Moreover, they expressed more concern over progressing to the next year of study (*P* = 0.029) but felt that their placements/learning opportunities were less severely impacted by travel restrictions (*P* = 0.004) and were less likely to agree that COVID-19 adversely affected their exam performance (*P* = 0.033). Lastly, they felt better supported regarding regular communication (*P* < 0.001), alternatives to clinical work (*P* = 0.032), question and answer opportunities with RCS staff (*P* = 0.005), online learning and teaching (*P* < 0.001), extra clinical skills learning opportunities (*P* = 0.006), and financial support (*P* < 0.001).

## Discussion

This multi-centre national study comprehensively investigated the impact of the COVID-19 pandemic on rural clinical placement experiences of students from 17 Australian universities to elucidate the impact of the pandemic on rural medical students’ clinical placements and learning quality and evaluate the support strategies implemented across rural Australia. Findings indicate that although students felt the COVID-19 pandemic negatively impacted their learning, they were generally satisfied with the academic, psychological, and social support provided by their RCS and approved of the adjusted learning methods implemented.

Although students expressed increased concerns about the quality of learning across all RCSs, the proportion concerned about their learning progression was relatively small. This suggests that the learning adaptations and support strategies implemented by the RCSs (Fig. 1) were helpful in mitigating student concerns about their learning experiences triggered by the pandemic. This is a positive reflection of the RCS program, demonstrating that RCSs across the country have been successful in responding to the pandemic.

Comparisons of the 2019 and 2020 FRAME survey data indicate an unexpected result, that students felt more supported by their RCS after commencement of the pandemic compared to the preceding year’s RCS cohort. This suggests that the disruptive impact of the pandemic may have triggered new ways in which RCS placement experiences can be enhanced. Internationally, the COVID-19 pandemic led to alternative means of academically and socially supporting students such as online discussion forums [20], peer mentoring [21, 22], virtual case-based learning [21], more frequent communication from staff [22], and virtual social support sessions for students [23]. As with the rest of the world, it will be vital for Australian RCSs to continue to evaluate the different learning and support strategies they have implemented (see Fig. 1), to determine which ones are important to sustain into the post-pandemic period. As noted previously, the pandemic has provided medical schools an opportunity to examine all aspects of their medical programs, and to review the suitability of curriculum for future doctors, so that the future medical workforce can be better trained and prepared to meet the health needs of communities they serve [2, 16, 24, 25].

Students who had completed only one RCS year expressed a need for more support when compared to students who had completed more than one RCS year. Whilst those with one RCS year’s experience may not have been new to clinical training, they were new to their placement community. This suggests that students undertaking RCS placements over two years had increased connectedness with the community, enabling increased resilience to some of the training uncertainty that resulted from the pandemic. In another COVID-19 study conducted in the United States, students that were newer to clinical learning environments had expressed greater concern around the impact of the pandemic on their medical education [26]. The literature also suggests that students that desire increased support may benefit from peer mentoring from more senior medical students, in addition to other learning and support strategies implemented by their medical schools [27].

Many differences emerged between the time of year students completed the survey, with those earlier in the year noting more negative experiences in relation to support questions. Mid-year respondents were likely in the midst of changes as RCSs were adjusting and trialling different learning and support strategies, whereas end-of-year respondents were more likely, with time, to be settled into adaptations, especially once the initial fear of the unknown passed. It is noted that experiences of the group that completed the survey mid-year relate to two specific RCSs only, but it is not possible to determine whether this cohort effect contributes to the observed differences.

As the pandemic continues to evolve and the medical education landscape continues to adapt with it, the lingering impacts of lockdowns, isolation while infectious, travel restrictions and the ‘new normal’ ways of teaching, learning, and living for medical students in rural locations requires monitoring. The mental health and wellbeing of these students also need to be watched. The importance of continuing provision of high-quality learning experiences for the future rural medical workforce cannot be underestimated.

## Limitations

This study reports a one-off survey that was conducted at the completion of extended RCS placements, but participants were not given any opportunity to further qualify their responses such as via open text. The survey will be re-administered 12 months later (with a different set of students) as a follow-up to the 2020 survey, to continue investigation of the ongoing impact of the COVID-19 pandemic on rural medical education in Australia. State comparisons were not permitted under the FRAME data sharing agreement to preserve anonymity, so this study could not investigate the possible impact of local acute events or varying pandemic severity and lockdown rules in different states on student learning experiences. The analysis of differences in survey timing is potentially confounded by differences between the RCSs (e.g., location of the university), not the timing itself.

## Conclusion

This national multi-centre survey of medical student experiences across most of rural Australia during the pandemic has provided evidence of both the impact on rural students’ clinical placements and learning quality as well as the positive impact of the various learning and support strategies and adaptations implemented by the RCSs across Australia. Students overall felt safe and well-supported academically, psychologically, and socially. RCSs will need to continue to evaluate and strengthen the positive strategies implemented during the pandemic that resulted in greater student support. This study has highlighted the need to provide more support to students who are newer to rural training environments. Future research could incorporate qualitative and longitudinal studies to further understanding of the continued impacts of the pandemic on rural medical education and the pandemic-trained generation of the medical workforce.

## Data Availability

Data are protected by ethics. All reasonable requests to access de-identified data can be made in writing to the corresponding author.

## Abbreviations

FRAME: Federation of Rural Australian Medical Educators
RCS: Rural Clinical School
